# Recessive/dominant model: Alternative choice in case-control-based genome-wide association studies

**DOI:** 10.1371/journal.pone.0254947

**Published:** 2021-07-21

**Authors:** Han-Ming Liu, Jin-Ping Zheng, Dan Yang, Zhao-Fa Liu, Zi Li, Zhen-Zhen Hu, Ze-Nan Li

**Affiliations:** School of Mathematics and Computer Science, Gannan Normal University, Ganzhou, China; University of Politehnica of Bucharest, ROMANIA

## Abstract

An additive genetic model is usually employed in case-control-based genome-wide association studies. The model usually encodes "AA", "Aa" and "aa" ("a" represents the minor allele) as three different numbers, implying the contribution of genotype "Aa" to the phenotype is different from "AA" and "aa". From the perspective of biological phenomena, the coding is reasonable since the phenotypes of lives are not "black and white". A case-control based study, however, has only two phenotypes, case and control, which means that the phenotypes are "black and white". It suggests that a recessive/dominant model may be an alternative to the additive model. In order to investigate whether the alternative is feasible, we conducted comparative experiments on several models used in those studies through chi-square test and logistic regression. Our simulation experiments demonstrate that a recessive model is better than the additive model. The area under the curve of the former has increased by 5% compared with the latter, the discrimination of identifying risk single nucleotide polymorphisms has been improved by 61%, and the precision has also reached 1.10 times that of the latter. Furthermore, the real data experiments show that the precision and area under the curve of the former are 16% and 20% higher than the latter respectively, and the area under the curve of dominant model of the former is 13% higher than the latter. The results indicate a recessive/dominant model may be an alternative to the additive model and suggest a new route for case-control-based studies.

## Introduction

The single nucleotide polymorphisms (SNPs) may lead to changes in individual characteristics or phenotypes, resulting in changes in disease risk or physiological characteristics [1]. How to explore the association between genes and diseases from the changes in genomes is a meaningful work. Ozaki K. et al. published such study firstly in 2002, which explored the association between functional SNPs in lymphotoxin-α gene and the susceptibility to myocardial infarction [2]. After this study, a landmark genome-wide association study (GWAS) was born in 2005—Klein R.J. et al. investigated a group of patients with age-related macular degeneration (AMD) and found two SNPs with significantly altered allele frequency compared to the healthy controls [3]. GWAS tested hundreds of thousands to millions of genetic variants in the human genomes to identify genotype-phenotype associations and has revolutionized the field of genetics of complex diseases in the past decade [4,5]. Since the publication of AMD GWAS, GWASes have achieved great success [6–8], and more than 50,000 significant genome-wide associations between genetic variants and common diseases/traits have been reported [9]. The emergence of International HapMap Project [10] and Biobank [11] promoted the development of GWAS further. As of 2017, more than 3,000 human GWAS had tested more than 1,800 diseases and traits, and revealed thousands of SNP associations [12]. The most commonly used method for GWAS is the case-control setup, which compares two large groups of individuals, a case group affected by a disease and a healthy control group. Early statistical power calculations indicated that this method might be better than linkage studies at detecting weak genetic effects [13].

There are usually three genetic models of genes: additive model (AM), dominant model (DM), and recessive model (RM). From AMD research in 2005 to the most recent study of genetic variants in infant and early childhood growth [3,14–22], the most commonly used model in GWAS is additive [23,24]. The genotypes "AA", "Aa" and "aa" ("a" is the minor allele) are coded as three different numbers in a genome dataset with AM. The coding implies that the contribution of genotype "Aa" to phenotype is different from "AA" and "aa". It is reasonable since the phenotypes of lives are not "black and white". But, for a case-control-based GWAS, the phenotype of an individual is either case or control, which indicates that the genotypes in the study are "black and white". Thus, we believe that RM/DM may be an alternative to AM in case-control-based GWASes. Our comparison experiment of simulation and real data show that RM/DM can better represent the phenotypic manifestations of case-control-based GWAS datasets than AM. And it has higher area under the curve (AUC), precision, discrimination and accuracy.

## Materials and methods

### Simulation data

Simulation data are used for quantitative analysis to evaluate the performance of all models to detect associations between diseases and genes. This study used PLINK 1.07 [25] to generate simulation data. A total of two scenarios were simulated:

**Scenario 1:** A total of 500 independent datasets were simulated. Each dataset consists of 1,000 cases, 1,000 controls, and 10,000 SNPs (100 of which are disease SNPs). The generating parameters of the datasets were combined by 10 odds ratios (ORs) (= 1.1, 1.2,…, 2.0) and 10 minor allele frequencies (MAFs) (= 0.05, 0.10,…, 0.5), that is, 100 combinations in total. For each combination, the data generation was repeated five times.

**Scenario 2:** The numbers of samples and MAFs are same as in scenario 1. Instead of specifying ORs, the penetrances of "AA" and "aa" were set to 0.01 and 0.1 respectively, and that of "Aa" was changed from 0.01 to 0.1 with step of 0.01. Thus, it includes a total of 100 combinations of MAFs and penetrances. Similarly, the data simulation was repeated five times for each combination, and thus produced 500 datasets.

### Real data

This study employed a real coronary artery disease (CAD) dataset to test the performance of RM, DM and AM models in identifying disease risk SNPs on real datasets. The dataset comes from one of the WTCCC1 research datasets of the Wellcome Trust Case Control Consortium (WTCCC) [26], which is a case-control dataset with 1,988 cases and 1,500 controls, containing 490,032 SNPs of the 22 autosomes and 10,536 SNPs of the sex chromosome X. We pre-processed the data to ensure its quality, including MAF test, Hardy-Weinberg equilibrium test, allele deletion test for each SNP, and SNP deletion test for each individual. The thresholds of the tests are 0.05, 0.01, 0.05 and 0.05, respectively. In addition, the SNPs in the exclusion list provided by WTCCC had also been removed. Only the 22 autosomes were used in our experiments. After pre-processing, the CAD dataset contains a total of 363,590 valid SNPs.

### Approaches

#### Tools of data generation and pre-processing

The simulation data in scenario 1 were generated by PLINK 1.07, and the data in scenario 2 were generated by a modified PLINK 1.07. We modified the PLINK to add a new generation mode implemented by specifying the penetrances of genotypes, since the original PLINK does not support this function. The modified PLINK is available on https://github.com/spvm2000/mPLINK. The pre-processing including data formats transformation was implemented by coPLINK [27].

#### Analysis approaches

The essence of GWAS is to explore the associations in phenotype-SNP data, and identify the SNPs with a score greater than the threshold as risky. This study employed the classic and basic chi-square test and logistic regression to calculate the association scores between phenotypes and SNPs to evaluate the performance of the models in identifying disease risk SNPs in simulation and real data. The scores of the two approaches produce *P* values which are negatively correlated with SNP risk. In order to transfer positive correlations from negative, the score of chi-square and logistic were subjected to an operation −log10(•).

#### T test

For the purpose of indicating the AUC differences of the models are statistically significant, we performed a two-tailed t test on the comparison results of AUCs through the analysis tool set of Microsoft Excel.

### Evaluation measurements

A discrimination odds ratio (*dOR*) and precision ratio (*PR*) were defined, and AUC was employed as the evaluation measurements for this study to compare the performance of all models in identifying disease risk SNPs.

#### Discrimination and discrimination odds ratio

If a genetic disease is caused by a gene mutation, it means that the base sequence of the gene has been partially or completely changed compared to a normal individual (that is, the gene has a risk SNP). In other words, there is a difference in genotype between the case and the control, or there is a difference in the degrees of association between the genotypes and phenotype of the risk SNPs and non-risk SNPs. Moreover, the association degrees of the risk SNPs are usually stronger than those of the non-risk SNPs. Here, we defined a measurement named as "discrimination" (represented by the letter "*d*") to describe this difference, which is defined as the ratio of the average score of the risk SNPs to that of the non-risk SNPs, namely,

$$d=Odds=\frac{\bar{{\mathrm{Score}}_{risk}}}{\bar{{\mathrm{Score}}_{null}}}(d\in [0,+\infty )),$$ (1)

where, $\bar{{\mathrm{Score}}_{risk}}$ represents the average score of the disease risk SNPs and $\bar{{\mathrm{Score}}_{null}}$ is the average score of the non-risk SNPs. When the scores (or the absolute values of the scores) are positively correlated with the degrees of associations, the larger the *d*, the greater the discrimination, indicating that the greater the difference in the associations between the two types of SNPs and the disease, is more conducive to identifying risk SNPs. Therefore, the discrimination of a model can represent the model’s capability to identify disease risk SNPs. In order to compare the discriminations of two models (or methods), we also defined a measurement called "discrimination odds ratio" (*dOR*), which is defined as follows:

$$dOR=\frac{d_{1}}{d_{2}}(dOR\in [0,+\infty )),$$ (2)

where, *d*_1_ and *d*_2_ are the discriminations of the two models/methods, respectively. Obviously, under the premise of that the discrimination is positively related to the association, *dOR* > 1 indicates that the model/method 1 is better than the model/method 2.

#### Precision ratio

Similarly, we define the precision ratio (*PR*) as:

$$PR=\frac{{\mathrm{Precision}}_{1}}{{\mathrm{Precision}}_{2}}(PR\in [0,+\infty )),$$ (3)

where, *Precision*_1_ and *Precision*_2_ represent the identifying precisions of the two models/methods, respectively. In this way, we can infer whether the identifying precision of model/method 1 is better than model/method 2 according to whether the *PR* is greater than 1.

#### AUC

AUC is defined as the area under a receiver operating characteristic curve (ROC) and is often used as an important evaluation measurement for model/method comparison in machine learning [28]. Here, we employed AUC to compare the performance of models AM, RM and DM in identifying risk SNPs.

## Experiments

### Experiments of AUC

We calculated the true positive rates (TPRs) and the false positive rates (FPRs) of each simulation dataset firstly, and then calculated their means. Finally, we plotted the ROCs based on these means and calculated the AUCs from the ROCs.

### Experiments of discrimination odds ratio over simulation datasets

The experiments calculated *dOR* means of all simulation datasets for scenario 1 and 2 on the non-additive models to AM of all approaches.

### Experiments of precision ratio over simulation datasets

The *P* value of chi-square and logistic were corrected by BH (Benjamini & Hochberg), and a *P*-cutoff method with a cutoff of 0.05 was applied to infer the significance of the SNPs. Similar to the experiments of discrimination odds ratio, we calculated the *PR* means of the simulation datasets for the scenarios on the non-additive models to AM of the approaches.

### Experiments of real data

In this experiment, RM, DM and AM models were used to identify risk SNPs, and BH correction was made to the *P* value of chi-square and logistic. In order to evaluate the disease risk SNPs identified by the approaches, we queried the SNP database [29] of the National Center for Biotechnology Information (NCBI) to obtain the genes where the SNPs are located. In addition, we consulted literatures to determine whether the genes are at risk of disease and infer the risk of the SNPs.

## Results

### Comparison over AUC

**Fig 1:**
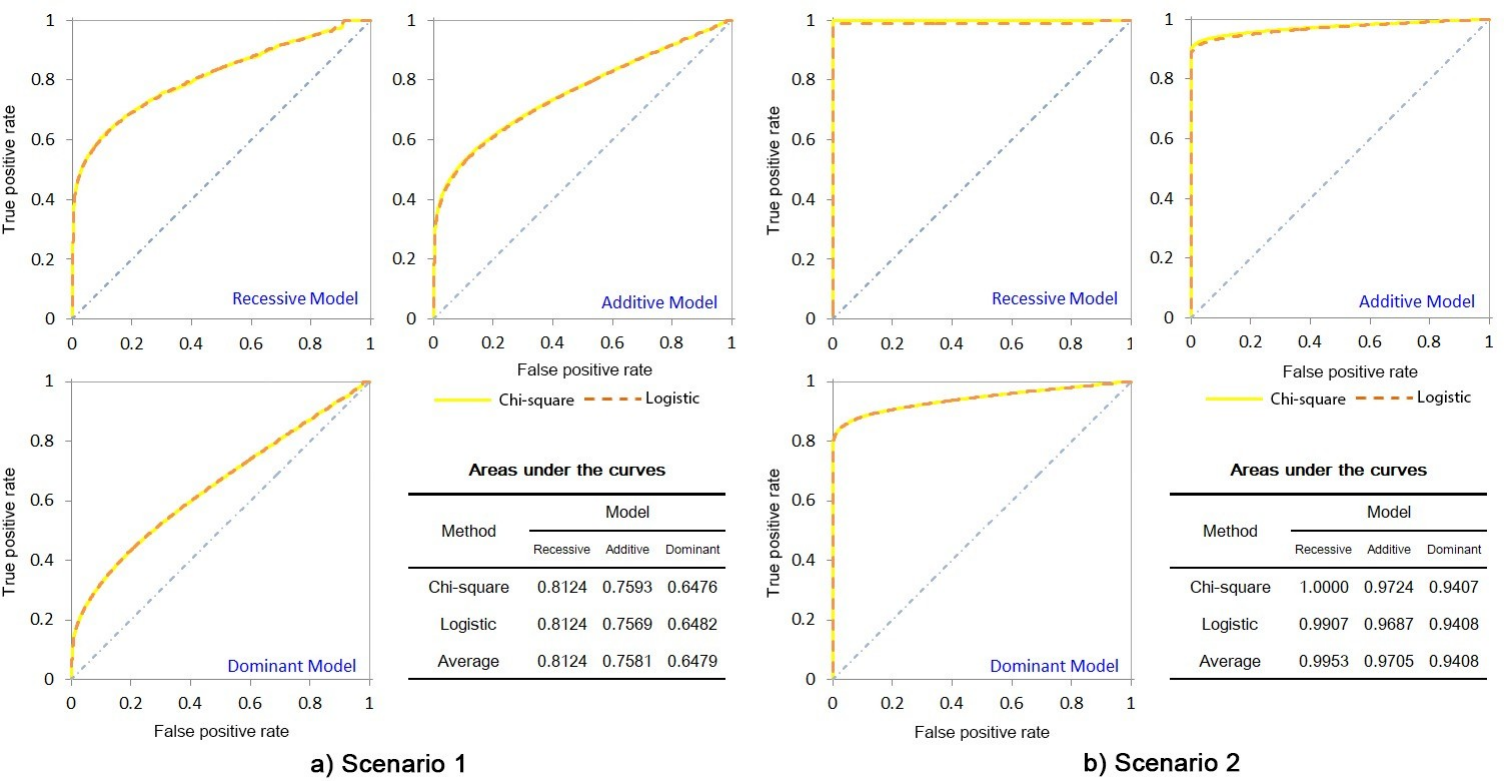
Receiver operating characteristic curves and areas under the curves on simulation datasets. Four significant figures are given here in order to display the area differences among the approaches.

Fig 1A and 1B show the three models’ ROC curves derived from the two approaches and the AUC lists calculated from the ROCs based on the datasets generated from simulation scenario 1 and 2, respectively.

Fig 1 intuitively shows that the RM’s ROC curves of the two approaches are almost overlap, and the curves are obviously more skewed to the upper left corner than AM, which means that the accuracy of RM is higher than AM. The figure also shows DM is weaker than AM. The AUCs on Fig 1 indicate that the AUC of RM is significantly higher than AM (average 0.8124 vs. 0.7581 and 0.9953 vs. 0.9705, respectively), and DM is greatly worse than AM (average 0.6479 vs. 0.7581 and 0.9408 vs. 0.9705, respectively). To test whether the differences are statistically significant, we tested them by a two-tailed t test. The *P* values of RM vs. AM in scenario 1 and 2 are 3.84×10^−32^ and 2.12×10^−10^, respectively, and those of DM vs. AM in the scenarios are 2.00×10^−81^ and 1.72×10^−17^, respectively. From the figure, we can calculate that the grand average AUC of RM and AM in the two scenarios are 0.9039 and 0.8643, that is, the AUC of RM is 5% higher than that of AM.

### Performance of identifying risk SNPs comparison over simulation datasets

Suppose the SNP scores (or the absolute values of the scores) are positively correlated to the degrees of association, then the discrimination odds ratio *dOR* > 1 indicates that the discrimination of model/method 1 is better than model/method 2. *PR* is similar. Table 1 shows the *dOR*s and *PR*s of the non-additive models to AM of the approaches on the simulation datasets. From the table, we can calculate the grand average *dOR* of RM and DM to AM are 1.61 and 0.78 respectively, which indicates that the risk SNP discrimination of RM is 1.61 times that of AM, that is, RM is significantly better than AM in identifying risk SNPs.

**Table 1:** *dOR*s and *PR*s on simulation datasets.

Approach	Scenario 1				Scenario 2			
	RM vs. AM		DM vs. AM		RM vs. AM		DM vs. AM	
	*dOR*	*PR*	*dOR*	*PR*	*dOR*	*PR*	*dOR*	*PR*
Chi-square	1.38	1.14	0.68	0.41	1.85	1.08	0.84	0.90
Logistic	1.45	1.16	0.70	0.45	1.74	1.01	0.89	0.92
Average^#^	1.42	1.15	0.69	0.43	1.80	1.05	0.87	0.91

^#^The grand average *dOR* of RM vs. AM and DM vs. AM in the two scenarios are 1.61 and 0.78, respectively. And, their grand average *PR*s are 1.10 and 0.67, respectively.

Furthermore, Table 1 shows the grand average *PR* of RM to AM is 1.10, which means that the average risk SNP identifying precision of RM is 10% higher than that of AM. Meanwhile, the table shows the grand average *PR* of DM to AM is 0.67, which indicates the identifying precision of DM is weaker than AM.

### Comparison over real dataset

The real dataset CAD was analyzed based on AM, RM and DM. With the *P*-cutoff of 0.05, we obtained the CAD risk SNP counts after querying SNP database on NCBI and consulting literatures, as shown in Table 2. The detailed results are listed in S1-S4 Tables in S1 File. Table 2 shows that the average *PR* of RM to AM is 1.16, which is higher than 1.00 of DM to AM (*P* value with two-tailed t test is 2.13×10^−2^). It indicates that RM is better than AM and DM on the CAD dataset.

**Table 2:** Risk SNP counts and *PR*s on CAD.

Approach	RM	DM	AM	*PR*	
				RM vs. AM	DM vs. AM
Chi-Square	35 (72)^#^	36 (86)	58 (141)	1.18	1.02
Logistic	31 (64)	36 (86)	63 (147)	1.13	0.98
Average	33 (68)	36 (86)	60.5 (144)	1.16	1.00

^#^ The numbers in the parentheses are the risk SNP counts based on the *P* threshold of 0.05.

**Fig 2:**
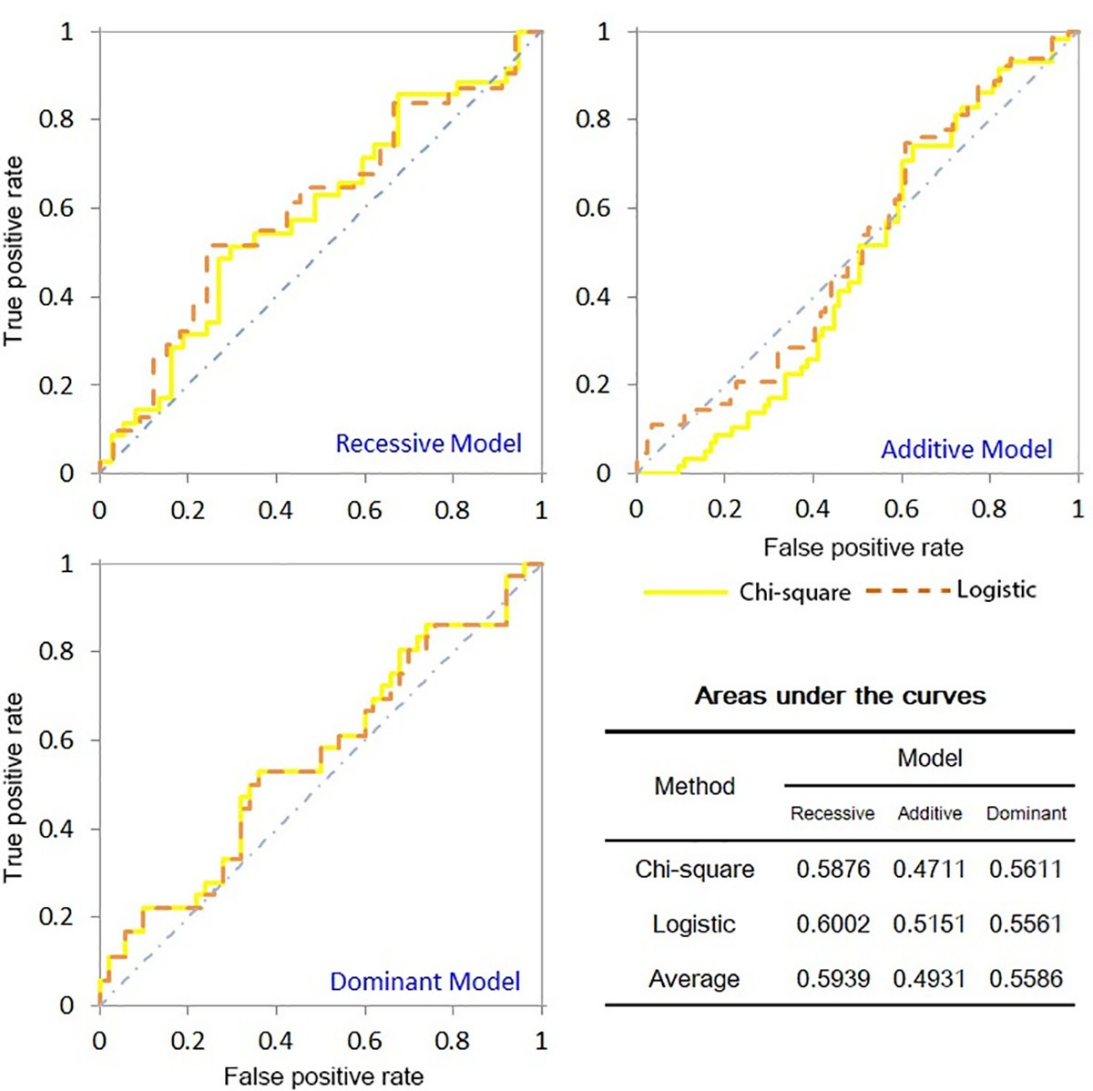
Receiver operating characteristic curves and areas under the curves on real dataset.

Based on the results of the real dataset, we obtained the three models’ ROC curves derived from the two approaches and the AUC list calculated from the ROCs (Fig 2).

From Fig 2, we can learn that the AUC of RM and DM are markedly higher than AM (20% and 13% higher respectively).

## Discussion

In the field of genetics, it is a high probability event that an improper genetic model assumption will lead to improper conclusions. Although AM is usually used in case-control-based GWAS, the dataset simplifies the phenotypes as two types, which means RM/DM may be an alternative to AM.

The comparative experiment of chi-square test and logistic regression based on simulation and real data verified this conjecture. The experiments show that RM is better than AM in terms of AUC, discrimination and precision. The real data experiments show that the AUC of DM is higher than that of RM, too. Existing works also approve this conjecture. In the study on risk of obesity and type 2 diabetes, Andrew R. Wood et al. identified two risk loci on obesity and type 2 diabetes, FTO and CDKAL1 [24] by employing a RM model. For the purpose of investigating whether novel nsCL/P risk loci could be identified by analyzing dominant/recessive genetic effects in SNP data from GWASes, the study of Bohmer A.C. et al. show that 18 loci are significant in DM and six loci are significant in RM among the 24 candidate loci, although they could not observe a new variant [30].

Our experiments show that RM is better than AM overall. Moreover, the real data experiments indicate the AUC of RM and DM are both higher than AM. The results suggest that AM is not necessarily choice in a case-control-based GWAS. How to evaluate AM, RM or DM is more suitable? The four-model strategy by Horita N. and Kaneko T. is a useful reference [31]. In addition, Bagos and Pantelis G. reviewed various methods and provided useful inspiration for us to genetic model selection in GWAS [32]. Moreover, it is a good idea that Kwak and Minjung used asymptotic property of the suptest to maintain the robustness of the Cochran–Armitage trend test when the genetic model is unknown [33].

## Supporting information

S1 File (DOCX)
