# Supplementary material for: Recessive/dominant model: Alternative choice in case-control-based genome-wide association studies
Source: PLoS One. 2021 Jul 21;16(7):e0254947. doi: 10.1371/journal.pone.0254947 (PMC8294554; doi:10.1371/journal.pone.0254947)
Supplement: S1 File — (DOCX) [file pone.0254947.s001.docx]

**Supplemental Information**

## Risk SNPs and their genes on real CAD

Tables S1 ~ S4 list all CAD disease risk SNPs identified by RM and AM for chi-square and logistic approaches. In order to assess whether these risk SNPs affect CAD, we consulted a total of 2292 articles, 95 of which indicate one of the genes is related to CAD risks. In the 95 works, 14 belong to GWAS. The models used in the GWASes include additive[25,36,56,66,77], dominant[76], all models (to select the best results after analyzing all the results)[24] and unspecified model[3,5,54,55,57,67,72], and the works employed various statistical analyses such as chi-square, linear regression and logistic regression. In the non-GWASes, many methods were used to identify the risk genes, such as pathological analysis, proteomic analysis, and electrocardiogram analysis.

Table S1. **Risk SNPs and their genes identified by chi-square in AM**

| **No.** | **Chr.** | **SNP Label** | **Gene** | **Relation** |
| --- | --- | --- | --- | --- |
| 1 | 1 | rs948620 | *None* | N |
| 2 | 1 | rs41499051 | *None* | N |
| 3 | 1 | rs41468647 | *DNM3* | Y[1-3] |
| 4 | 1 | rs41528947 | *CCDC18-AS1* | N |
| 5 | 2 | rs1522103 | *C2orf70* | N |
| 6 | 3 | rs41400446 | *None* | N |
| 7 | 4 | rs41426946 | *PPA2* | Y[4] |
| 8 | 5 | rs41505353 | *SPOCK1* | Y[5] |
| 9 | 5 | rs41482053 | *SEMA5A* | Y[6] |
| 10 | 5 | rs41421845 | *LINC02107* | N |
| 11 | 6 | rs6929834 | *None* | N |
| 12 | 6 | rs41342244 | *LOC105378102* | N |
| 13 | 8 | rs41414254 | *None* | N |
| 14 | 8 | rs41431045 | *None* | N |
| 15 | 9 | rs41416444 | *None* | N |
| 16 | 11 | rs4106126 | *GRM5* | N |
| 17 | 12 | rs41416849 | *None* | N |
| 18 | 14 | rs41324646 | *FRMD6/FRMD6-AS2* | Y[7] |
| 19 | 14 | rs41521248 | *None* | N |
| 20 | 16 | rs41334049 | *None* | N |
| 21 | 16 | rs41337248 | *KIAA0513* | Y[8] |
| 22 | 16 | rs3888264 | *CNGB1* | Y[9] |
| 23 | 18 | rs17068201 | *CDH20* | N |
| 24 | 20 | rs41384844 | *RIN2* | Y[10] |
| 25 | 20 | rs41419652 | *None* | N |
| 26 | 21 | rs7281940 | *None* | N |
| 27 | 12 | rs10850052 | *None* | N |
| 28 | 22 | rs9617611 | *BCL2L13* | Y[11] |
| 29 | 3 | rs41497550 | *MECOM/LOC105374205* | Y[12] |
| 30 | 12 | rs41514645 | *SNP not found* |  |
| 31 | 19 | rs17380748 | *None* | N |
| 32 | 8 | rs17075115 | *LOC105377795* | N |
| 33 | 14 | rs41502454 | *LRFN5* | Y[13] |
| 34 | 3 | rs41412751 | *KLHL6* | N |
| 35 | 14 | rs41339746 | *DNAL1* | Y[14] |
| 36 | 4 | rs41505154 | *EXOSC9* | Y[15] |
| 37 | 6 | rs41509944 | *None* | N |
| 38 | 16 | rs17569609 | *GRIN2A* | Y[12,16] |
| 39 | 17 | rs9890438 | *KRTAP3-2* | Y[17] |
| 40 | 3 | rs41419044 | *None* | N |
| 41 | 4 | rs41369347 | *CXXC4-AS1* | N |
| 42 | 12 | rs17019941 | *LINC01619* | N |
| 43 | 9 | rs1333049 | *None* | N |
| 44 | 3 | rs41338146 | *LOC105374069* | N |
| 45 | 9 | rs1333048 | *None* | N |
| 46 | 7 | rs41500551 | *None* | N |
| 47 | 15 | rs41363546 | *KIF7* | Y[18] |
| 48 | 16 | rs3785143 | *SLC6A2* | Y[19] |
| 49 | 7 | rs41526251 | *None* | N |
| 50 | 4 | rs41472746 | *None* | N |
| 51 | 9 | rs2891168 | *CDKN2B-AS1* | Y[20-24] |
| 52 | 9 | rs4977574 | *CDKN2B-AS1* | Y[20-24] |
| 53 | 5 | rs41436848 | *MAML1* | Y[25-27] |
| 54 | 3 | rs41352751 | *SETD5* | Y[28-30] |
| 55 | 15 | rs41439544 | *LINC01578/CHD2* | Y[31] |
| 56 | 10 | rs34243358 | *None* | N |
| 57 | 9 | rs10965224 | *CDKN2B-AS1* | Y[20-24] |
| 58 | 9 | rs6475606 | *CDKN2B-AS1* | Y[20-24] |
| 59 | 9 | rs1333042 | *CDKN2B-AS1* | Y[20-24] |
| 60 | 12 | rs6489847 | *None* | N |
| 61 | 1 | rs41319344 | *None* | N |
| 62 | 9 | rs10757272 | *CDKN2B-AS1* | Y[20-24] |
| 63 | 9 | rs9632884 | *CDKN2B-AS1* | Y[20-24] |
| 64 | 18 | rs41510648 | *DCC* | Y[32] |
| 65 | 3 | rs41450044 | *ADAMTS9-AS2* | N |
| 66 | 18 | rs17084348 | *None* | N |
| 67 | 8 | rs16889862 | *None* | N |
| 68 | 22 | rs41459445 | *HMGXB4* | N |
| 69 | 5 | rs41444348 | *None* | N |
| 70 | 11 | rs41324851 | *LRRC4C* | Y[33] |
| 71 | 21 | rs1556329 | *COL18A1* | Y[34] |
| 72 | 21 | rs41353548 | *COL6A2* | Y[35] |
| 73 | 5 | rs41514444 | *FCHO2* | Y[36] |
| 74 | 21 | rs2255764 | *MX2* | Y[37] |
| 75 | 6 | rs1385225 | *LOC105377839* | N |
| 76 | 11 | rs1365404 | *None* | N |
| 77 | 9 | rs10965219 | *CDKN2B-AS1* | Y[20-24] |
| 78 | 1 | rs41496444 | *None* | N |
| 79 | 8 | rs41326949 | *LOC107986931* | N |
| 80 | 5 | rs41466348 | *PDE4D* | Y[38-43] |
| 81 | 3 | rs41401648 | *IGSF10* | Y[44] |
| 82 | 17 | rs9908572 | *None* | N |
| 83 | 5 | rs12514672 | *PRLR* | Y[45] |
| 84 | 3 | rs41455050 | *FHIT /LOC107986015* | Y[46] |
| 85 | 5 | rs41523345 | *CDH18* | Y[47] |
| 86 | 2 | rs7571463 | *None* | N |
| 87 | 10 | rs17155289 | *None* | N |
| 88 | 16 | rs4780746 | *GRIN2A* | Y[12,16] |
| 89 | 14 | rs41406645 | *LRRC9* | N |
| 90 | 10 | rs17782733 | *VSTM4* | Y[48] |
| 91 | 9 | rs7865618 | *CDKN2B-AS1* | Y[20-24] |
| 92 | 4 | rs17531757 | *ATP8A1* | Y[49] |
| 93 | 14 | rs41521556 | *LOC105370653 /LOC105370655* | N |
| 94 | 16 | rs16958951 | *None* | N |
| 95 | 1 | rs41515244 | *DAB1* | Y[50] |
| 96 | 8 | rs41368244 | *None* | N |
| 97 | 9 | rs10965215 | *CDKN2B-AS1* | Y[20-24] |
| 98 | 10 | rs17119894 | *None* | N |
| 99 | 5 | rs41404844 | *None* | N |
| 100 | 10 | rs12772945 | *None* | N |
| 101 | 11 | rs41464948 | *MAP6* | Y[51] |
| 102 | 9 | rs7049105 | *CDKN2B-AS1* | Y[20-24] |
| 103 | 9 | rs10965212 | *CDKN2B-AS1* | Y[20-24] |
| 104 | 20 | rs41396046 | *SNP not found* |  |
| 105 | 2 | rs1133353 | *CAPN10-DT* | N |
| 106 | 9 | rs438156 | *LOC105375999* | N |
| 107 | 9 | rs564398 | *CDKN2B-AS1* | Y[20-24] |
| 108 | 1 | rs10492990 | *None* | N |
| 109 | 5 | rs30530 | *SEPTIN8* | Y[52] |
| 110 | 10 | rs10905409 | *LOC105376398* | N |
| 111 | 17 | rs16961474 | *None* | N |
| 112 | 12 | rs41341248 | *LOC105369791* | N |
| 113 | 7 | rs34100060 | *CREB3L2* | Y[53] |
| 114 | 1 | rs2133189 | *MIA3* | Y[54-57] |
| 115 | 5 | rs565928 | *None* | N |
| 116 | 5 | rs436495 | *None* | N |
| 117 | 5 | rs383830 | *None* | N |
| 118 | 1 | rs17465637 | *MIA3* | Y[54-57] |
| 119 | 5 | rs1490855 | *None* | N |
| 120 | 4 | rs41339646 | *None* | N |
| 121 | 1 | rs10908445 | *None* | N |
| 122 | 6 | rs2306915 | *SYNE1* | Y[58-62] |
| 123 | 1 | rs41403751 | *None* | N |
| 124 | 3 | rs1979845 | *SLC22A13* | Y[63] |
| 125 | 10 | rs1779028 | *None* | N |
| 126 | 6 | rs1474787 | *MTHFD1L* | Y[56,64-66] |
| 127 | 5 | rs41523246 | *None* | N |
| 128 | 4 | rs41398444 | *None* | N |
| 129 | 5 | rs6868877 | *None* | N |
| 130 | 5 | rs449650 | *None* | N |
| 131 | 6 | rs6922269 | *MTHFD1L* | Y[56,64-66] |
| 132 | 5 | rs6875635 | *None* | N |
| 133 | 22 | rs688034 | *SEZ6L* | Y[67,68] |
| 134 | 4 | rs2272160 | *SNORA24* | N |
| 135 | 5 | rs4244387 | *None* | N |
| 136 | 20 | rs10485636 | *None* | N |
| 137 | 5 | rs4400148 | *None* | N |
| 138 | 11 | rs600256 | *None* | N |
| 139 | 6 | rs9478448 | *None* | N |
| 140 | 5 | rs10900893 | *None* | N |
| 141 | 16 | rs237179 | *None* | N |

Note: The "None" in column Gene represents the SNP does not lie in any gene.

### Table S2. Risk SNPs and their genes identified by chi-square in RM

| **No.** | **Chr.** | **SNP Label** | **Gene** | **Relation** |
| --- | --- | --- | --- | --- |
| 1 | 1 | rs41499051 | *None* | N |
| 2 | 1 | rs41319344 | *None* | N |
| 3 | 3 | rs41401648 | *IGSF10* | Y[44] |
| 4 | 3 | rs41497550 | *MECOM/LOC105374205* | Y[12] |
| 5 | 3 | rs41352751 | *SETD5* | Y[28-30] |
| 6 | 3 | rs41412751 | *KLHL6* | N |
| 7 | 5 | rs41436848 | *MAML1* | Y[25-27] |
| 8 | 6 | rs41509944 | *None* | N |
| 9 | 8 | rs41343444 | *CPQ* | N |
| 10 | 8 | rs41431045 | *None* | N |
| 11 | 10 | rs34243358 | *None* | N |
| 12 | 12 | rs16926425 | *SOX5* | Y[69-72] |
| 13 | 14 | rs41521556 | *LOC105370653 /LOC105370655* | N |
| 14 | 16 | rs41334049 | *None* | N |
| 15 | 19 | rs17380748 | *None* | N |
| 16 | 20 | rs41419652 | *None* | N |
| 17 | 22 | rs41459445 | *HMGXB4* | N |
| 18 | 21 | rs41353548 | *COL6A2* | Y[35] |
| 19 | 21 | rs1556329 | *COL18A1* | Y[34] |
| 20 | 1 | rs41499648 | *NAV1* | Y[73] |
| 21 | 16 | rs41337248 | *KIAA0513* | Y[8] |
| 22 | 3 | rs41400446 | *None* | N |
| 23 | 4 | rs41369347 | *CXXC4-AS1* | N |
| 24 | 4 | rs41505154 | *EXOSC9* | Y[15] |
| 25 | 6 | rs41342244 | *LOC105378102* | N |
| 26 | 5 | rs41505353 | *SPOCK1* | Y[5] |
| 27 | 15 | rs41363546 | *KIF7* | Y[18] |
| 28 | 4 | rs41354944 | *None* | N |
| 29 | 9 | rs41416444 | *None* | N |
| 30 | 14 | rs41324646 | *FRMD6 /FRMD6-AS2* | Y[7] |
| 31 | 12 | rs41416849 | *None* | N |
| 32 | 2 | rs41450346 | *None* | N |
| 33 | 15 | rs41439544 | *LINC01578 /CHD2* | Y[31] |
| 34 | 14 | rs41502454 | *LRFN5* | Y[13] |
| 35 | 7 | rs12700671 | *None* | N |
| 36 | 2 | rs16849921 | *LOC107985981* | N |
| 37 | 20 | rs41384844 | *RIN2* | Y[10] |
| 38 | 5 | rs41404844 | *None* | N |
| 39 | 18 | rs17068201 | *CDH20* | N |
| 40 | 1 | rs948620 | *None* | N |
| 41 | 7 | rs41420446 | *JAZF1* | Y[74,75] |
| 42 | 9 | rs1333049 | *None* | N |
| 43 | 6 | rs41443144 | *None* | N |
| 44 | 1 | rs41515244 | *DAB1* | Y[50] |
| 45 | 9 | rs1333048 | *None* | N |
| 46 | 5 | rs41523345 | *CDH18* | Y[47] |
| 47 | 4 | rs41426946 | *PPA2* | Y[4] |
| 48 | 5 | rs11740402 | *PDE4D* | Y[38-43] |
| 49 | 9 | rs10965224 | *CDKN2B-AS1* | Y[20-24] |
| 50 | 9 | rs6475606 | *CDKN2B-AS1* | Y[20-24] |
| 51 | 3 | rs11924705 | *None* | N |
| 52 | 16 | rs41473349 | *FTO* | Y[76-78] |
| 53 | 8 | rs41451349 | *None* | N |
| 54 | 6 | rs41502946 | *SNP not found* |  |
| 55 | 3 | rs41462744 | *CCDC80* | Y[79] |
| 56 | 16 | rs16958951 | *None* | N |
| 57 | 9 | rs1333042 | *CDKN2B-AS1* | Y[20-24] |
| 58 | 9 | rs2891168 | *CDKN2B-AS1* | Y[20-24] |
| 59 | 2 | rs41464947 | *MYO1B* | Y[80] |
| 60 | 2 | rs41373446 | *RAB10* | Y[81] |
| 61 | 3 | rs41450044 | *ADAMTS9-AS2* | N |
| 62 | 9 | rs4977574 | *CDKN2B-AS1* | Y[20-24] |
| 63 | 13 | rs1442701 | *None* | N |
| 64 | 3 | rs41419044 | *None* | N |
| 65 | 5 | rs41482053 | *SEMA5A* | Y[6] |
| 66 | 5 | rs41444348 | *None* | N |
| 67 | 9 | rs9632884 | *CDKN2B-AS1* | Y[20-24] |
| 68 | 4 | rs41398444 | *None* | N |
| 69 | 16 | rs7203379 | *HYDIN* | Y[82] |
| 70 | 9 | rs10757272 | *CDKN2B-AS1* | Y[20-24] |
| 71 | 14 | rs41406645 | *LRRC9* | N |
| 72 | 22 | rs688034 | *SEZ6L* | Y[67,68] |

Note: The "None" in column Gene represents the SNP does not lie in any gene.

### Table S3. Risk SNPs and their genes identified by logistic in AM

| **No.** | **Chr.** | **SNP Label** | **Gene** | **Relation** |
| --- | --- | --- | --- | --- |
| 1 | 1 | rs948620 | *None* | N |
| 2 | 1 | rs41499051 | *None* | N |
| 3 | 1 | rs41468647 | *DNM3* | Y[1-3] |
| 4 | 1 | rs41528947 | *CCDC18-AS1* | N |
| 5 | 2 | rs1522103 | *C2orf70* | N |
| 6 | 3 | rs41400446 | *None* | N |
| 7 | 4 | rs41426946 | *PPA2* | Y[4] |
| 8 | 5 | rs41505353 | *SPOCK1* | Y[5] |
| 9 | 5 | rs41482053 | *SEMA5A* | Y[6] |
| 10 | 5 | rs41421845 | *LINC02107* | N |
| 11 | 6 | rs6929834 | *None* | N |
| 12 | 6 | rs41342244 | *LOC105378102* | N |
| 13 | 8 | rs41414254 | *None* | N |
| 14 | 8 | rs41431045 | *None* | N |
| 15 | 9 | rs41416444 | *None* | N |
| 16 | 11 | rs4106126 | *GRM5* | N |
| 17 | 12 | rs41416849 | *None* | N |
| 18 | 14 | rs41324646 | *FRMD6 /FRMD6-AS2* | Y[7] |
| 19 | 14 | rs41521248 | *None* | N |
| 20 | 16 | rs41334049 | *None* | N |
| 21 | 16 | rs41337248 | *KIAA0513* | Y[8] |
| 22 | 16 | rs3888264 | *CNGB1* | Y[9] |
| 23 | 18 | rs17068201 | *CDH20* | N |
| 24 | 20 | rs41384844 | *RIN2* | Y[10] |
| 25 | 20 | rs41419652 | *None* | N |
| 26 | 21 | rs7281940 | *None* | N |
| 27 | 12 | rs10850052 | *None* | N |
| 28 | 3 | rs41497550 | *MECOM /LOC105374205* | Y[12] |
| 29 | 22 | rs9617611 | *BCL2L13* | Y[11] |
| 30 | 12 | rs41514645 | *SNP not found* |  |
| 31 | 3 | rs41412751 | *KLHL6* | N |
| 32 | 4 | rs41505154 | *EXOSC9* | Y[15] |
| 33 | 14 | rs41502454 | *LRFN5* | Y[13] |
| 34 | 19 | rs17380748 | *None* | N |
| 35 | 6 | rs41509944 | *None* | N |
| 36 | 14 | rs41339746 | *DNAL1* | Y[14] |
| 37 | 8 | rs17075115 | *LOC105377795* | N |
| 38 | 16 | rs17569609 | *GRIN2A* | Y[12,16] |
| 39 | 3 | rs41338146 | *LOC105374069* | N |
| 40 | 12 | rs17019941 | *LINC01619* | N |
| 41 | 9 | rs1333049 | *None* | N |
| 42 | 17 | rs9890438 | *KRTAP3-2* | Y[17] |
| 43 | 4 | rs41369347 | *CXXC4-AS1* | N |
| 44 | 3 | rs41419044 | *None* | N |
| 45 | 9 | rs1333048 | *None* | N |
| 46 | 7 | rs41526251 | *None* | N |
| 47 | 7 | rs41500551 | *None* | N |
| 48 | 15 | rs41363546 | *KIF7* | Y[18] |
| 49 | 4 | rs41472746 | *None* | N |
| 50 | 9 | rs2891168 | *CDKN2B-AS1* | Y[20-24] |
| 51 | 9 | rs4977574 | *CDKN2B-AS1* | Y[20-24] |
| 52 | 5 | rs41436848 | *MAML1* | Y[25-27] |
| 53 | 16 | rs3785143 | *SLC6A2* | Y[19] |
| 54 | 3 | rs41352751 | *SETD5* | Y[28-30] |
| 55 | 15 | rs41439544 | *LINC01578 /CHD2* | Y[31] |
| 56 | 1 | rs41319344 | *None* | N |
| 57 | 9 | rs10965224 | *CDKN2B-AS1* | Y[20-24] |
| 58 | 9 | rs6475606 | *CDKN2B-AS1* | Y[20-24] |
| 59 | 10 | rs34243358 | *None* | N |
| 60 | 9 | rs1333042 | *CDKN2B-AS1* | Y[20-24] |
| 61 | 12 | rs6489847 | *None* | N |
| 62 | 18 | rs41510648 | *DCC* | Y[32] |
| 63 | 9 | rs10757272 | *CDKN2B-AS1* | Y[20-24] |
| 64 | 9 | rs9632884 | *CDKN2B-AS1* | Y[20-24] |
| 65 | 3 | rs41450044 | *ADAMTS9-AS2* | N |
| 66 | 8 | rs16889862 | *None* | N |
| 67 | 5 | rs41444348 | *None* | N |
| 68 | 18 | rs17084348 | *None* | N |
| 69 | 11 | rs41324851 | *LRRC4C* | Y[33] |
| 70 | 22 | rs41459445 | *HMGXB4* | N |
| 71 | 21 | rs41353548 | *COL6A2* | Y[35] |
| 72 | 21 | rs2255764 | *MX2* | Y[37] |
| 73 | 5 | rs41514444 | *FCHO2* | Y[36] |
| 74 | 21 | rs1556329 | *COL18A1* | Y[34] |
| 75 | 6 | rs1385225 | *LOC105377839* | N |
| 76 | 17 | rs9908572 | *None* | N |
| 77 | 11 | rs1365404 | *None* | N |
| 78 | 3 | rs41401648 | *IGSF10* | Y[44] |
| 79 | 9 | rs10965219 | *CDKN2B-AS1* | Y[20-24] |
| 80 | 2 | rs7571463 | *None* | N |
| 81 | 1 | rs41496444 | *None* | N |
| 82 | 8 | rs41326949 | *LOC107986931* | N |
| 83 | 5 | rs12514672 | *PRLR* | Y[45] |
| 84 | 16 | rs4780746 | *GRIN2A* | Y[12,16] |
| 85 | 5 | rs41523345 | *CDH18* | Y[47] |
| 86 | 10 | rs17782733 | *VSTM4* | Y[48] |
| 87 | 14 | rs41406645 | *LRRC9* | N |
| 88 | 5 | rs41466348 | *PDE4D* | Y[38-43] |
| 89 | 14 | rs41521556 | *LOC105370653 /LOC105370655* | N |
| 90 | 4 | rs17531757 | *ATP8A1* | Y[49] |
| 91 | 1 | rs41515244 | *DAB1* | Y[50] |
| 92 | 10 | rs17155289 | *None* | N |
| 93 | 3 | rs41455050 | *FHIT /LOC107986015* | Y[46] |
| 94 | 9 | rs7865618 | *CDKN2B-AS1* | Y[20-24] |
| 95 | 8 | rs41368244 | *None* | N |
| 96 | 9 | rs10965215 | *CDKN2B-AS1* | Y[20-24] |
| 97 | 16 | rs16958951 | *None* | N |
| 98 | 10 | rs17119894 | *None* | N |
| 99 | 10 | rs12772945 | *None* | N |
| 100 | 5 | rs41404844 | *None* | N |
| 101 | 20 | rs41396046 | *SNP not found* |  |
| 102 | 11 | rs41464948 | *MAP6* | Y[51] |
| 103 | 1 | rs10492990 | *None* | N |
| 104 | 9 | rs7049105 | *CDKN2B-AS1* | Y[20-24] |
| 105 | 9 | rs564398 | *CDKN2B-AS1* | Y[20-24] |
| 106 | 7 | rs34100060 | *CREB3L2* | Y[53] |
| 107 | 9 | rs10965212 | *CDKN2B-AS1* | Y[20-24] |
| 108 | 2 | rs1133353 | *CAPN10-DT* | N |
| 109 | 9 | rs438156 | *LOC105375999* | N |
| 110 | 12 | rs41341248 | *LOC105369791* | N |
| 111 | 5 | rs565928 | *None* | N |
| 112 | 5 | rs383830 | *None* | N |
| 113 | 5 | rs436495 | *None* | N |
| 114 | 5 | rs41523246 | *None* | N |
| 115 | 1 | rs10908445 | *None* | N |
| 116 | 1 | rs2133189 | *MIA3* | Y[54-57] |
| 117 | 5 | rs30530 | *SEPTIN8* | Y[52] |
| 118 | 10 | rs10905409 | *LOC105376398* | N |
| 119 | 6 | rs2306915 | *SYNE1* | Y[58-62] |
| 120 | 17 | rs16961474 | *None* | N |
| 121 | 4 | rs41398444 | *None* | N |
| 122 | 11 | rs600256 | *None* | N |
| 123 | 20 | rs10485636 | *None* | N |
| 124 | 1 | rs17465637 | *MIA3* | Y[54-57] |
| 125 | 6 | rs9478448 | *None* | N |
| 126 | 4 | rs41339646 | *None* | N |
| 127 | 10 | rs1779028 | *None* | N |
| 128 | 5 | rs1490855 | *None* | N |
| 129 | 16 | rs237179 | *None* | N |
| 130 | 6 | rs1474787 | *MTHFD1L* | Y[56,64-66] |
| 131 | 3 | rs262983 | *YEATS2* | Y[83] |
| 132 | 5 | rs6868877 | *None* | N |
| 133 | 5 | rs449650 | *None* | N |
| 134 | 5 | rs4244387 | *None* | N |
| 135 | 5 | rs6875635 | *None* | N |
| 136 | 1 | rs41403751 | *None* | N |
| 137 | 1 | rs6586490 | *None* | N |
| 138 | 10 | rs4948583 | *LINC00841* | Y[84] |
| 139 | 20 | rs348800 | *None* | N |
| 140 | 16 | rs10852376 | *MYH11* | Y[85-88] |
| 141 | 3 | rs1979845 | *SLC22A13* | Y[63] |
| 142 | 6 | rs6922269 | *MTHFD1L* | Y[56,64-66] |
| 143 | 5 | rs4400148 | *None* | N |
| 144 | 22 | rs688034 | *SEZ6L* | Y[67,68] |
| 145 | 5 | rs10900893 | *None* | N |
| 146 | 5 | rs10475585 | *ADAM19* | Y[89-95] |
| 147 | 9 | rs7044859 | *CDKN2B-AS1* | Y[20-24] |

Note: The "None" in column Gene represents the SNP does not lie in any gene.

### Table S4. Risk SNPs and their genes identified by logistic in RM

| **No.** | **Chr.** | **SNP Label** | **Gene** | **Relation** |
| --- | --- | --- | --- | --- |
| 1 | 1 | rs41499051 | *None* | N |
| 2 | 1 | rs41319344 | *None* | N |
| 3 | 3 | rs41401648 | *IGSF10* | Y[44] |
| 4 | 3 | rs41497550 | *MECOM /LOC105374205* | Y[12] |
| 5 | 3 | rs41352751 | *SETD5* | Y[28-30] |
| 6 | 3 | rs41412751 | *KLHL6* | N |
| 7 | 5 | rs41436848 | *MAML1* | Y[25-27] |
| 8 | 6 | rs41509944 | *None* | N |
| 9 | 8 | rs41343444 | *CPQ* | N |
| 10 | 8 | rs41431045 | *None* | N |
| 11 | 10 | rs34243358 | *None* | N |
| 12 | 12 | rs16926425 | *SOX5* | Y[69-72] |
| 13 | 14 | rs41521556 | *LOC105370653 /LOC105370655* | N |
| 14 | 19 | rs17380748 | *None* | N |
| 15 | 20 | rs41419652 | *None* | N |
| 16 | 22 | rs41459445 | *HMGXB4* | N |
| 17 | 21 | rs41353548 | *COL6A2* | Y[35] |
| 18 | 16 | rs41337248 | *KIAA0513* | Y[8] |
| 19 | 1 | rs41499648 | *NAV1* | Y[73] |
| 20 | 4 | rs41369347 | *CXXC4-AS1* | N |
| 21 | 4 | rs41505154 | *EXOSC9* | Y[15] |
| 22 | 15 | rs41363546 | *KIF7* | Y[18] |
| 23 | 6 | rs41342244 | *LOC105378102* | N |
| 24 | 4 | rs41354944 | *None* | N |
| 25 | 5 | rs41505353 | *SPOCK1* | Y[5] |
| 26 | 12 | rs41416849 | *None* | N |
| 27 | 16 | rs41334049 | *None* | N |
| 28 | 9 | rs41416444 | *None* | N |
| 29 | 2 | rs41450346 | *None* | N |
| 30 | 15 | rs41439544 | *LINC01578 /CHD2* | Y[31] |
| 31 | 3 | rs41400446 | *None* | N |
| 32 | 14 | rs41502454 | *LRFN5* | Y[13] |
| 33 | 14 | rs41324646 | *FRMD6 /FRMD6-AS2* | Y[7] |
| 34 | 2 | rs16849921 | *LOC107985981* | N |
| 35 | 5 | rs41404844 | *None* | N |
| 36 | 7 | rs41420446 | *JAZF1* | Y[74,75] |
| 37 | 1 | rs948620 | *None* | N |
| 38 | 9 | rs1333049 | *None* | N |
| 39 | 6 | rs41443144 | *None* | N |
| 40 | 9 | rs1333048 | *None* | N |
| 41 | 1 | rs41515244 | *DAB1* | Y[50] |
| 42 | 5 | rs41523345 | *CDH18* | Y[47] |
| 43 | 20 | rs41384844 | *RIN2* | Y[10] |
| 44 | 4 | rs41426946 | *PPA2* | Y[4] |
| 45 | 18 | rs17068201 | *CDH20* | N |
| 46 | 9 | rs10965224 | *CDKN2B-AS1* | Y[20-24] |
| 47 | 9 | rs6475606 | *CDKN2B-AS1* | Y[20-24] |
| 48 | 3 | rs11924705 | *None* | N |
| 49 | 16 | rs41473349 | *FTO* | Y[76-78] |
| 50 | 8 | rs41451349 | *None* | N |
| 51 | 3 | rs41462744 | *CCDC80* | Y[79] |
| 52 | 6 | rs41502946 | *SNP not found* |  |
| 53 | 9 | rs1333042 | *CDKN2B-AS1* | Y[20-24] |
| 54 | 9 | rs2891168 | *CDKN2B-AS1* | Y[20-24] |
| 55 | 2 | rs41464947 | *MYO1B* | Y[80] |
| 56 | 2 | rs41373446 | *RAB10* | Y[81] |
| 57 | 3 | rs41450044 | *ADAMTS9-AS2* | N |
| 58 | 9 | rs4977574 | *CDKN2B-AS1* | Y[20-24] |
| 59 | 3 | rs41419044 | *None* | N |
| 60 | 5 | rs41444348 | *None* | N |
| 61 | 9 | rs9632884 | *CDKN2B-AS1* | Y[20-24] |
| 62 | 5 | rs41482053 | *SEMA5A* | Y[6] |
| 63 | 4 | rs41398444 | *None* | N |
| 64 | 9 | rs10757272 | *CDKN2B-AS1* | Y[20-24] |

Note: The "None" in column Gene represents the SNP does not lie in any gene.

## References

1. Loebel DA, Tsoi B, Wong N, Tam PP (2005) A conserved noncoding intronic transcript at the mouse Dnm3 locus. Genomics 85: 782-789.

2. Aurora AB, Mahmoud AI, Luo X, Johnson BA, van Rooij E, et al. (2012) MicroRNA-214 protects the mouse heart from ischemic injury by controlling Ca(2)(+) overload and cell death. J Clin Invest 122: 1222-1232.

3. Trinh J, Gustavsson EK, Vilariño-Güell C, Bortnick S, Latourelle J, et al. (2016) DNM3 and genetic modifiers of age of onset in LRRK2 Gly2019Ser parkinsonism: a genome-wide linkage and association study. The Lancet Neurology 15: 1248-1256.

4. Vasilescu C, Ojala TH, Brilhante V, Ojanen S, Hinterding HM, et al. (2018) Genetic Basis of Severe Childhood-OnsetáCardiomyopathies. Journal of the American College of Cardiology 72: 2324-2338.

5. Nolan DK, Sutton B, Haynes C, Johnson J, Sebek J, et al. (2012) Fine mapping of a linkage peak with integration of lipid traits identifies novel coronary artery disease genes on chromosome 5. Bmc Genetics 13.

6. Jin Z, Chau MD, Bao ZZ (2006) Sema3D, Sema3F, and Sema5A are expressed in overlapping and distinct patterns in chick embryonic heart. Dev Dyn 235: 163-169.

7. Narumiya H, Hidaka K, Shirai M, Terami H, Aburatani H, et al. (2007) Endocardiogenesis in embryoid bodies: novel markers identified by gene expression profiling. Biochem Biophys Res Commun 357: 896-902.

8. Vafiadaki E, Sanoudou D, Arvanitis DA, Catino DH, Kranias EG, et al. (2007) Phospholamban interacts with HAX-1, a mitochondrial protein with anti-apoptotic function. J Mol Biol 367: 65-79.

9. Tripathi ON (2011) Cardiac Ion Channels and Heart Rate and Rhythm. Heart Rate and Rhythm: Springer. pp. 3-31.

10. Carney DS, Davies BA, Horazdovsky BF (2006) Vps9 domain-containing proteins: activators of Rab5 GTPases from yeast to neurons. Trends Cell Biol 16: 27-35.

11. Otsu K, Murakawa T, Yamaguchi O (2015) BCL2L13 is a mammalian homolog of the yeast mitophagy receptor Atg32. Autophagy 11: 1932-1933.

12. Ciaccio C, Tucci A, Scuvera G, Estienne M, Esposito S, et al. (2017) 16p13 microduplication without CREBBP involvement: Moving toward a phenotype delineation. European journal of medical genetics 60: 159-162.

13. Johnston KJ, Adams MJ, Nicholl BI, Ward J, Strawbridge RJ, et al. (2019) Identification of novel common variants associated with chronic pain using conditional false discovery rate analysis with major depressive disorder and assessment of pleiotropic effects of LRFN5. Translational Psychiatry 9: 1-10.

14. Vincent JB (2018) Dynein axonemal light chain 4: Involvement in congenital mirror movement disorder. Dyneins: Elsevier. pp. 436-449.

15. Xi Z, Jun-Hui S, Ling-Jing J, Fei T (2019) Advance in Serum Biomarkers for Early Identification of Cerebral Infarction and Intracerebral Hemorrhage. Chin J Stroke 14.

16. Coll M, Striano P, Ferrer-Costa C, Campuzano O, Matés J, et al. (2017) Targeted next-generation sequencing provides novel clues for associated epilepsy and cardiac conduction disorder/SUDEP. PloS one 12: e0189618.

17. Moon S-H, Kim Y-J, Kim Y-K, Kim D-J, Lee J-Y, et al. (2011) Genome-wide survey of copy number variants associated with blood pressure and body mass index in a Korean population. Genomics & Informatics 9: 152-160.

18. Yu HN (2006) Characterization of kinesin superfamily4 (kif4) and kinesin superfamily7 (kif7) in teleost development: City University of Hong Kong.

19. Verschure DO, Baas F, van Eck-Smit BL, Somsen GA, Verberne HJ (2018) Polymorphism of SLC6A2 gene does not influence outcome of myocardial 123 I-mIBG scintigraphy in patients with chronic heart failure. Journal of Nuclear Cardiology 25: 900-906.

20. AbdulAzeez S, Al-Nafie A, Al-Shehri A, Borgio J, Baranova E, et al. (2016) Intronic polymorphisms in the CDKN2B-AS1 gene are strongly associated with the risk of myocardial infarction and coronary artery disease in the Saudi population. International journal of molecular sciences 17: 395.

21. Greco S, Zaccagnini G, Perfetti A, Fuschi P, Valaperta R, et al. (2016) Long noncoding RNA dysregulation in ischemic heart failure. Journal of translational medicine 14: 183.

22. Huang K, Zhong J, Li Q, Zhang W, Chen Z, et al. (2019) Effects of CDKN2B‐AS1 polymorphisms on the susceptibility to coronary heart disease. Molecular genetics & genomic medicine: e955.

23. Thomas AA, Feng B, Chakrabarti S (2017) ANRIL regulates production of extracellular matrix proteins and vasoactive factors in diabetic complications. American Journal of Physiology-Endocrinology and Metabolism 314: E191-E200.

24. Schaefer AS, Richter GM, Nothnagel M, Laine ML, Noack B, et al. (2010) COX-2 is associated with periodontitis in Europeans. Journal of Dental Research 89: 384-388.

25. Mezzavilla M, Iorio A, Bobbo M, D'Eustacchio A, Merlo M, et al. (2014) Insight into genetic determinants of resting heart rate. Gene 545: 170-174.

26. Preuss C, Capredon M, Wünnemann F, Chetaille P, Prince A, et al. (2016) Family based whole exome sequencing reveals the multifaceted role of notch signaling in congenital heart disease. PLoS genetics 12: e1006335.

27. Zhang J, Niu J, Tian B, Zhao M (2019) microRNA‐193b protects against myocardial ischemia‐reperfusion injury in mouse by targeting mastermind‐like 1. Journal of cellular biochemistry.

28. Pinard A, Guey S, Guo D, Cecchi AC, Kharas N, et al. (2019) The pleiotropy associated with de novo variants in CHD4, CNOT3, and SETD5 extends to moyamoya angiopathy. Genetics in Medicine: 1-5.

29. Osipovich AB, Gangula R, Vianna PG, Magnuson MA (2016) Setd5 is essential for mammalian development and the co-transcriptional regulation of histone acetylation. Development 143: 4595-4607.

30. Szczałuba K, Brzezinska M, Kot J, Rydzanicz M, Walczak A, et al. (2016) SETD5 loss‐of‐function mutation as a likely cause of a familial syndromic intellectual disability with variable phenotypic expression. American Journal of Medical Genetics Part A 170: 2322-2327.

31. Kulkarni S, Nagarajan P, Wall J, Donovan DJ, Donell RL, et al. (2008) Disruption of chromodomain helicase DNA binding protein 2 (CHD2) causes scoliosis. American Journal of Medical Genetics Part A 146: 1117-1127.

32. Li Q, Wang P, Ye K, Cai H (2015) Central role of SIAH inhibition in DCC-dependent cardioprotection provoked by netrin-1/NO. Proceedings of the National Academy of Sciences 112: 899-904.

33. Li P, Xu G, Li G, Wu M (2014) Function and mechanism of tumor suppressor gene LRRC4/NGL-2. Molecular cancer 13: 266.

34. Peloso GM, Auer PL, Bis JC, Voorman A, Morrison AC, et al. (2014) Association of low-frequency and rare coding-sequence variants with blood lipids and coronary heart disease in 56,000 whites and blacks. The American Journal of Human Genetics 94: 223-232.

35. Grossman TR, Gamliel A, Wessells RJ, Taghli-Lamallem O, Jepsen K, et al. (2011) Over-expression of DSCAM and COL6A2 cooperatively generates congenital heart defects. PLoS genetics 7: e1002344.

36. Sabater-Lleal M, Huffman JE, de Vries PS, Marten J, Mastrangelo MA, et al. (2019) Genome-Wide Association Transethnic Meta-Analyses Identifies Novel Associations Regulating Coagulation Factor VIII and von Willebrand Factor Plasma Levels. Circulation 139: 620-635.

37. Clancy RM, Markham AJ, Jackson T, Rasmussen SE, Blumenberg M, et al. (2017) Cardiac fibroblast transcriptome analyses support a role for interferogenic, profibrotic, and inflammatory genes in anti-SSA/Ro-associated congenital heart block. American Journal of Physiology-Heart and Circulatory Physiology 313: H631-H640.

38. Wang Q, Liu Y, Fu Q, Xu B, Zhang Y, et al. (2017) Inhibiting insulin-mediated β2-adrenergic receptor activation prevents diabetes-associated cardiac dysfunction. Circulation 135: 73-88.

39. Shi Q, Li M, Mika D, Fu Q, Kim S, et al. (2017) Heterologous desensitization of cardiac β-adrenergic signal via hormone-induced βAR/arrestin/PDE4 complexes. Cardiovascular research 113: 656-670.

40. Nastina O, Pleskach G, Kursina N, Bazyka O, Makarevich O, et al. (2016) Structural and functional state of heart left ventricle depending on polymorphism rs966221 phosphodiesterase 4D gene in emergency workers of the Chornobyl NPP suffering from coronary heart disease. Problemy radiatsiinoi medytsyny ta radiobiolohii 21: 312-335.

41. Sakai M, Suzuki T, Tomita K, Yamashita S, Palikhe S, et al. (2017) Diminished responsiveness to dobutamine as an inotrope in mice with cecal ligation and puncture-induced sepsis: attribution to phosphodiesterase 4 upregulation. American Journal of Physiology-Heart and Circulatory Physiology 312: H1224-H1237.

42. Martin TP, Hortigon-Vinagre MP, Findlay JE, Elliott C, Currie S, et al. (2014) Targeted disruption of the heat shock protein 20–phosphodiesterase 4D (PDE4D) interaction protects against pathological cardiac remodelling in a mouse model of hypertrophy. FEBS Open Bio 4: 923-927.

43. Gretarsdottir S, Thorleifsson G, Reynisdottir ST, Manolescu A, Jonsdottir S, et al. The gene encoding phosphodiesterase 4D confers risk of ischemic stroke. Nature Genetics.

44. Salzano A, Marra AM, Proietti M, Raparelli V, Heaney LM (2019) Biomarkers in heart failure and associated diseases. Disease markers 2019.

45. Schennink A, Trott JF, Manjarin R, Lemay DG, Freking BA, et al. (2015) Comparative genomics reveals tissue-specific regulation of prolactin receptor gene expression. Journal of molecular endocrinology 54: 1-15.

46. Dannewitz Prosseda S, Tian X, Kuramoto K, Boehm M, Sudheendra D, et al. (2019) FHIT, a novel modifier gene in pulmonary arterial hypertension. American journal of respiratory and critical care medicine 199: 83-98.

47. Chen C-P, Chang S-Y, Lin C-J, Chern S-R, Wu P-S, et al. (2018) Prenatal diagnosis of a familial 5p14. 3-p14. 1 deletion encompassing CDH18, CDH12, PMCHL1, PRDM9 and CDH10 in a fetus with congenital heart disease on prenatal ultrasound. Taiwanese Journal of Obstetrics and Gynecology 57: 734-738.

48. Hsu CY, Salazar MG, Miller S, Meyers C, Ding C, et al. (2019) Comparison of human tissue microarray to human pericyte transcriptome yields novel perivascular cell markers. Stem Cells and Development 28: 1214-1223.

49. Yan H, Li Y, Wang C, Zhang Y, Liu C, et al. (2017) Contrary microRNA expression pattern between fetal and adult cardiac remodeling: Therapeutic value for heart failure. Cardiovascular toxicology 17: 267-276.

50. Herrero-Turrión MJ, Velasco A, Arevalo R, Aijón J, Lara JM (2010) Characterisation and differential expression during development of a duplicate Disabled-1 (Dab1) gene from zebrafish. Comparative Biochemistry and Physiology Part B: Biochemistry and Molecular Biology 155: 217-229.

51. Csont T, Murlasits Z, Ménesi D, Kelemen JZ, Bencsik P, et al. (2015) Tissue-specific gene expression in rat hearts and aortas in a model of vascular nitrate tolerance. Journal of cardiovascular pharmacology 65: 485-493.

52. Shiryaev A, Kostenko S, Dumitriu G, Moens U (2012) Septin 8 is an interaction partner and in vitro substrate of MK5. World journal of biological chemistry 3: 98.

53. Ferlosio A, Doldo E, Polisca P, Orlandi A (2013) Low-grade fibromyxoid sarcoma: an unusual cardiac location. Cardiovascular Pathology 22: e15-e17.

54. Shahid SU, Shabana N, Rehman A, Humphries S (2018) GWAS implicated risk variants in different genes contribute additively to increase the risk of coronary artery disease (CAD) in the Pakistani subjects. Lipids in health and disease 17: 89.

55. Shahid SU, Cooper JA, Beaney KE, Li K, Rehman A, et al. (2017) Genetic risk analysis of coronary artery disease in Pakistani subjects using a genetic risk score of 21 variants. Atherosclerosis 258: 1-7.

56. Angelakopoulou A, Shah T, Sofat R, Shah S, Berry DJ, et al. (2011) Comparative analysis of genome-wide association studies signals for lipids, diabetes, and coronary heart disease: Cardiovascular Biomarker Genetics Collaboration. European heart journal 33: 393-407.

57. Consortium MIG (2009) Genome-wide association of early-onset myocardial infarction with single nucleotide polymorphisms and copy number variants. Nature genetics 41: 334.

58. Haskell GT, Jensen BC, Samsa LA, Marchuk D, Huang W, et al. (2017) Whole exome sequencing identifies truncating variants in nuclear envelope genes in patients with cardiovascular disease. Circulation: Cardiovascular Genetics 10: e001443.

59. Wu L, Xiang B, Zhang H, He X, Shih C, et al. (2017) Three novel recessive mutations in LAMA2, SYNE1, and TTN are identified in a single case with congenital muscular dystrophy. Neuromuscular Disorders 27: 1018-1022.

60. Zhang Q, Bethmann C, Worth NF, Davies JD, Wasner C, et al. (2007) Nesprin-1 and-2 are involved in the pathogenesis of Emery–Dreifuss muscular dystrophy and are critical for nuclear envelope integrity. Human molecular genetics 16: 2816-2833.

61. Zhou C, Rao L, Warren DT, Shanahan CM, Zhang Q (2018) Mouse models of nesprin-related diseases. Biochemical Society Transactions 46: 669-681.

62. Szabadosova V, Boronova I, Ferenc P, Tothova I, Bernasovska J, et al. (2018) Analysis of selected genes associated with cardiomyopathy by next‐generation sequencing. Journal of clinical laboratory analysis 32: e22254.

63. Suh W (2013) Characterization and regulation of organic anion transporters: Rutgers University-Graduate School-New Brunswick.

64. Palmer BR, Slow S, Ellis KL, Pilbrow AP, Skelton L, et al. (2014) Genetic polymorphism rs6922269 in the MTHFD1L gene is associated with survival and baseline active vitamin B12 levels in post-acute coronary syndromes patients. PloS one 9: e89029.

65. Franceschini N, Carty C, Bůžková P, Reiner AP, Garrett T, et al. (2011) Association of genetic variants and incident coronary heart disease in multiethnic cohorts: the PAGE study. Circulation: Cardiovascular Genetics 4: 661-672.

66. Nilesh J. Samani ASH, Richard J. Dixon, Jennifer H. Barrett (2007) Genomewide Association Analysis of Coronary Artery Disease. New England Journal of Medicine 357: 443.

67. Makeeva O, Sleptsov A, Kulish E, Barbarash O, Mazur A, et al. (2015) Genomic study of cardiovascular continuum comorbidity. Acta Naturae (англоязычная версия) 7.

68. Shah T, Zabaneh D, Gaunt T, Swerdlow DI, Shah S, et al. (2013) Gene-centric analysis identifies variants associated with interleukin-6 levels and shared pathways with other inflammation markers. Circulation: Cardiovascular Genetics 6: 163-170.

69. Seifert MB, Olesen MS, Christophersen IE, Nielsen JB, Carlson J, et al. (2019) Genetic variants on chromosomes 7p31 and 12p12 are associated with abnormal atrial electrical activation in patients with early‐onset lone atrial fibrillation. Annals of Noninvasive Electrocardiology: e12661.

70. Husser D, Büttner P, Stübner D, Ueberham L, Platonov PG, et al. (2017) PR Interval Associated Genes, Atrial Remodeling and Rhythm Outcome of Catheter Ablation of Atrial Fibrillation—A Gene-Based Analysis of GWAS Data. Frontiers in genetics 8: 224.

71. Li A, Hooli B, Mullin K, Tate RE, Bubnys A, et al. (2017) Silencing of the Drosophila ortholog of SOX5 leads to abnormal neuronal development and behavioral impairment. Human molecular genetics 26: 1472-1482.

72. Kolek MJ, Edwards TL, Muhammad R, Balouch A, Shoemaker MB, et al. (2014) A genome-wide association study to identify genomic modulators of rate control therapy in patients with atrial fibrillation. The American journal of cardiology 114: 593-600.

73. Stroud DM, Yang T, Bersell K, Kryshtal DO, Nagao S, et al. (2016) Contrasting nav1. 8 activity in scn10a−/− ventricular myocytes and the intact heart. Journal of the American Heart Association 5: e002946.

74. Bae KB, Kim MO, Yu DH, Shin MJ, Kim HJ, et al. (2011) Overexpression of Jazf1 induces cardiac malformation through the upregulation of pro-apoptotic genes in mice. Transgenic research 20: 1019-1031.

75. Venturin M, Bentivegna A, Moroni R, Larizza L, Riva P (2005) Evidence by expression analysis of candidate genes for congenital heart defects in the NF1 microdeletion interval. Annals of human genetics 69: 508-516.

76. Gustavsson J, Mehlig K, Leander K, Berg C, Tognon G, et al. (2016) FTO gene variation, macronutrient intake and coronary heart disease risk: a gene–diet interaction analysis. European journal of nutrition 55: 247-255.

77. Gustavsson J, Mehlig K, Leander K, Lissner L, Björck L, et al. (2014) FTO genotype, physical activity, and coronary heart disease risk in Swedish men and women. Circulation: Cardiovascular Genetics 7: 171-177.

78. Mathiyalagan P, Adamiak M, Mayourian J, Sassi Y, Liang Y, et al. (2019) FTO-dependent N6-methyladenosine regulates cardiac function during remodeling and repair. Circulation 139: 518-532.

79. Sasagawa S, Nishimura Y, Sawada H, Zhang E, Okabe S, et al. (2016) Comparative transcriptome analysis identifies CCDC80 as a novel gene associated with pulmonary arterial hypertension. Frontiers in pharmacology 7: 142.

80. Mc Cormack A, Taylor J, Gregersen N, George AM, Love DR (2013) Delineation of 2q32q35 deletion phenotypes: two apparent “proximal” and “distal” syndromes. Case reports in genetics 2013.

81. Szablewski L (2017) Glucose transporters in healthy heart and in cardiac disease. International journal of cardiology 230: 70-75.

82. Liu Y, Cao Y, Li Y, Lei D, Li L, et al. (2018) Novel Genetic Variants of Sporadic Atrial Septal Defect (ASD) in a Chinese Population Identified by Whole-Exome Sequencing (WES). Medical science monitor: international medical journal of experimental and clinical research 24: 1340.

83. Yamada Y, Yasukochi Y, Kato K, Oguri M, Horibe H, et al. (2018) Identification of 26 novel loci that confer susceptibility to early‑onset coronary artery disease in a Japanese population. Biomedical reports 9: 383-404.

84. Dogan M, Beach S, Simons R, Lendasse A, Penaluna B, et al. (2018) Blood-Based Biomarkers for Predicting the Risk for Five-Year Incident Coronary Heart Disease in the Framingham Heart Study via Machine Learning. Genes 9: 641.

85. Wang C, Wang F, Cao Q, Li Z, Huang L, et al. (2018) The Effect of Mecp2 on Heart Failure. Cellular Physiology and Biochemistry 47: 2380-2387.

86. Isselbacher EM, Lino Cardenas CL, Lindsay ME (2016) Hereditary influence in thoracic aortic aneurysm and dissection. Circulation 133: 2516-2528.

87. LaHaye S, Corsmeier D, Bowman JL, Fitzgerald-Butt S, Zender G, et al. (2015) Utilization of Whole-Exome Sequencing to Identify Causative Mutations in Familial Congenital Heart Disease. Circulation 132: A12295-A12295.

88. Xia X-D, Zhou Z, Yu X-h, Zheng X-L, Tang C-K (2017) Myocardin: a novel player in atherosclerosis. Atherosclerosis 257: 266-278.

89. Qi B, Newcomer RG, Sang Q-XA (2009) ADAM19/adamalysin 19 structure, function, and role as a putative target in tumors and inflammatory diseases. Current pharmaceutical design 15: 2336-2348.

90. Chesneau V, Becherer JD, Zheng Y, Erdjument-Bromage H, Tempst P, et al. (2003) Catalytic properties of ADAM19. Journal of Biological Chemistry 278: 22331-22340.

91. Zhou H-M, Weskamp G, Chesneau V, Sahin U, Vortkamp A, et al. (2004) Essential role for ADAM19 in cardiovascular morphogenesis. Molecular and cellular biology 24: 96-104.

92. Kurohara K, Komatsu K, Kurisaki T, Masuda A, Irie N, et al. (2004) Essential roles of Meltrin β (ADAM19) in heart development. Developmental biology 267: 14-28.

93. Komatsu K, Wakatsuki S, Yamada S-i, Yamamura K-i, Miyazaki J-i, et al. (2007) Meltrin β expressed in cardiac neural crest cells is required for ventricular septum formation of the heart. Developmental biology 303: 82-92.

94. Horiuchi K, Zhou H-M, Kelly K, Manova K, Blobel CP (2005) Evaluation of the contributions of ADAMs 9, 12, 15, 17, and 19 to heart development and ectodomain shedding of neuregulins β1 and β2. Developmental biology 283: 459-471.

95. Goldmuntz E, Paluru P, Glessner J, Hakonarson H, Biegel JA, et al. (2011) Microdeletions and microduplications in patients with congenital heart disease and multiple congenital anomalies. Congenital heart disease 6: 592-602.
